# Defining the Environment in Organism–Environment Systems

**DOI:** 10.3389/fpsyg.2020.01285

**Published:** 2020-07-07

**Authors:** Amanda Corris

**Affiliations:** Department of Philosophy, University of Cincinnati, Cincinnati, OH, United States

**Keywords:** enactivism, ecological psychology, developmental systems theory, developmental niche, naturalization of perception

## Abstract

Enactivism and ecological psychology converge on the relevance of the environment in understanding perception and action. On both views, perceiving organisms are not merely passive receivers of environmental stimuli, but rather form a dynamic relationship with their environments in such a way that shapes how they interact with the world. In this paper, I suggest that while enactivism and ecological psychology enjoy a shared specification of the environment as the cognitive domain, on both accounts, the structure of the environment, itself, is unspecified beyond that of contingent relations with the species-typical sensorimotor capacities of perceiving organisms. This lack of specification creates a considerable gap in theory regarding the organization of organisms as coupled with their environments. I argue that this gap can be filled by drawing from resources in developmental systems theory, namely, specifying the environmental state-space as a developmental niche that shapes and is shaped by individual organisms over developmental and, on a population scale, evolutionary time. Defining the environment as an organism’s developmental niche makes it clearer how and why certain contingencies have arisen, in turn, strengthening a joint appeal to both enactivism and ecological psychology as theories asserting complementarity between organisms and their environments.

## Introduction

Enactivism and ecological psychology converge on the relevance of the environment in understanding perception and action. On both views, perceiving organisms are not merely passive receivers of environmental stimuli, but rather form a dynamic relationship with their environments in such a way that shapes how they interact with the world. Much of the attention in the shared literature between enactivism and ecological psychology has focused on the cognitive capacities of a perceiving organism in relation to its environment; less attention has been given to the environmental setting as a state-space, which is context-sensitive and organism-specific. As the environment plays a defining role in the sort of interactions that are possible for perceivers, specifying the structure of the environment for a species, or even a particular organism, can shed light on the nature of perception. The aim of this paper is to draw out similarities between enactivism and ecological psychology by specifying the structure of an organism’s particular environmental setting in such a way that illustrates how that structure partly organizes the organism–environment system and thus what features are perceptually relevant. A detailed account of the environment on an enactivism–ecological psychology framework can, in turn, provide guidance for a naturalized theory of perception. I suggest that viewing a perceiver’s environment as a developmental niche specifies the environment in an organism–environment system at the scale of the individual, thus providing a way of talking about how individual variation in perceptual abilities and traits can have an impact across developmental, behavioral, and evolutionary timescales.

In ‘Enacting a World’ I detail the ways in which the environment is discussed within the enactive literature. ‘Perceiving Environmental Information’ provides an overview of the concepts used within ecological psychology to describe the environment as guiding perception and action. In ‘Specifying the Cognitive Domain’ I suggest that specifying an organism’s cognitive domain as its developmental niche, as an integral part of a larger developmental system, can serve as a way of understanding organism–environment interaction as it is discussed in both the enactive and the ecological psychology literature. This conception of the environment, which draws on resources from the developmental systems theory (DST) can be built into a shared enactive-ecological psychology framework for an appropriately naturalized account of perception.

## Enacting a World

Enactive approaches to cognition share a commitment to a principle of dynamic coupling between organisms and their environments, with action being fundamentally guided by perception. Though emergent varieties of enactivism may differ in their philosophical aims (Ward et al., 2017), they each view the organism–environment relation as central to understanding the phenomenon of cognition. Additionally, they share a general commitment to rejecting computationalist, representationalist conceptions of cognition that posit it as a form of processing via symbol manipulation. For enactivists, a suitable explanation of cognition requires viewing it as a global process occurring as a result of dynamic interaction across multiple scales of organismal organization (with emphasis on the bodily scale, see also Chemero, 2009 for similar views) and the environmental state-space, rather than locally, as a matter of neural mechanisms.

On the conception of the enactive approach detailed in *The Embodied Mind* (Varela et al., 1991, see also Thompson, 2004), cognition emerges as a result of coupled interactions between organisms as autonomous systems and their environmental milieu. This relation is actualized through interactions between the organism via its sensorimotor capacities and the environmental features to which it is sensitive. Notably, not all environmental features play a constitutive role in an organism’s environmental milieu. The sensorimotor structure of the organism constrains which features are perceivable and thus actionable. Therefore, an organism’s embodiment plays a central role in constituting cognition, as “cognition depends upon the kinds of experience that come from having a body with various sensorimotor capacities” (Varela et al., 1991, 173). Humans lack the capacity to perceive ultraviolet light, and so ultraviolet light cannot modulate action for human perceivers. Honeybees, which enjoy the capacity to perceive ultraviolet light, regularly treat it as an action-guiding visual cue.

While humans and honeybees both share the same physical world, their perceived worlds drastically differ due to their variation in sensorimotor capacities. Thus, an organism *enacts* a perceived world depending on its sensorimotor capacities. As Varela et al. (1991) stress, “perception is not simply embedded within and constrained by the surrounding world; it also contributes to the enactment of this surrounding world” (174). Drawing on similar claims by Merleau-Ponty, they describe the organism as both initiating and shaping its environment, with both systems being “bound together in reciprocal specification and selection” (174). While an organism’s sensorimotor structure determines which environmental features are salient in its perceived world, the actual enactment of such a world is possible through the distinctive organization of organisms as living systems.

### The Organization of Living Systems

Early enactive work (Varela et al., 1974; Maturana and Varela, 1987) provided the foundation for understanding the organization of living beings. On this view, a defining feature of living beings is that they are continually self-producing—they are structured such that they are able to maintain themselves as a unit over time. This feature is referred to as *autopoiesis* (from Greek *auto*-, self, and *poiesis*, production). Autopoietic systems are specified as networks of processes with certain enabling relations. If these relations fail to hold, the system will necessarily disintegrate (Varela et al., 1974). The canonical example in this body of work is the cell. A cell can be conceived of as autopoietic system due to the way in which its internal processes enable the system to persist:

It is a network of chemical reactions which produce molecules such that (i) through their interactions generate and participate recursively in the same network of reactions which produced them, and (ii) realize the cell as a material entity. Thus the cell as a physical unity, topographically and operationally separable from the background, remains as such only insofar as this organization is continuously realized under permanent turnover of matter, regardless of its changes in form and specificity of its constitutive chemical reactions (Varela et al., 1974, 188).

Here, the environment is specified as merely the background in which the physical unity that is the cell is contrasted. The cell, as an autopoietic system, is “operationally separable” in that it undergoes a particular set of reactions that effectively forms an operationally closed network. Additionally, the cell’s membrane constitutes a boundary that distinguishes it as an entity from its environmental setting. In this context, autopoiesis captures metabolic self-production—it specifies the type of chemical reactions necessary for a living entity to maintain itself over time. For Maturana and Varela (1987), metabolic processes are central to a conceptualization of life, as they constitute the “dynamic transformations” that enable a living system to persist (Maturana and Varela, 1987, 46) and, as a result, form a membrane that serves as a spatial boundary for an individual cell.

The same organizational pattern can generally be found at the scale of larger organisms such as animals. Although these organisms may vary in structural form, they are organized in the same self-producing manner, in that they are “internally self-constructive in such a way as to regulate actively their interactions with their environments” (Thompson and Stapleton, 2009, 24). In other words, organisms are endowed with the ability to maintain their internal dynamics through self-regulation. An artifact of this organizational property is that it specifies an environmental state-space as well, described as the features that are operationally external to the organism’s self-regulatory capacities such that they are not necessary for the operational closure of the organism as a living system, though they may be necessary for its persisting over time.

Thus, a distinction can be drawn between two co-acting, yet organizationally distinguishable, systems. This property of the organism is referred to as its *autonomy* because it specifies the organism as a system that is “composed of processes that generate and sustain that system as a unity” (24). Because the internal, self-regulatory dynamics of the autonomous system are necessary for its persisting as a unity, it can be said to be operationally closed in the same manner that cells are. Importantly, as Thompson and Stapleton point out, “operational closure does not imply that conditions not belonging to the system cannot also be necessary” (24). Living systems are thermodynamically open, such that they undergo processes to regulate the flow of energy both between them (from the environment into the system) and within them (as regulatory processes internal to the system).

The properties of autonomy and operational closure can, in certain contexts, define a spatial boundary to a system as well. It is important to note that autonomous systems are not necessarily autopoietic systems because autonomous systems do not need to be spatially bound for their self-regulation. Thompson and Stapleton offer the example of a human or non-human animal social group as an autonomous system that is not spatially bound and therefore not autopoietic. As Froese et al. (2007) note:

It is generally claimed that autonomy in living systems is a feature of self-production or autopoiesis. However, this restriction of autonomy to living systems is unsatisfactory because we also want to refer to some systems as autonomous even though they are not characterized by metabolic self-production, for example artificial and social systems (Froese et al., 2007, 5; Luisi, 2003).

Further enactive work thus aims at a taxonomy of systems where autopoietic systems are members of a broader class of autonomous systems (Froese et al., 2007). Given Maturana and Varela’s (1987) specification of metabolic processes as those responsible for the dynamic transformation of components within the cell as a living system, drawing a distinction between types of structural arrangements that result in the same type of network can be helpful, namely, in the case of understanding social cognition as arising from interactions between two or more distinct systems.

### Specifying an Environment

According to the enactive approach to cognition, living systems are autonomous systems that are structured by their own internal, operationally closed regulatory dynamics as well as their thermodynamically open regulatory dynamics with the environment. Organisms engage in energy transfer from environment, but they do not do so entirely passively. Environmental features have a degree of valence for individual organisms. For a honeybee, the ultraviolet color pattern found in the center of a flower indicates a potential pollen location; for humans, the redness of a tomato indicates it is ready to be harvested and eaten. Organisms do not engage in passive reception of sensory stimuli but in positively or negatively valenced interactions with the environment. On an enactive view, this process is referred to as sense-making: it is “behavior or conduct in relation to environmental significance or valence, which the organism itself enacts or brings forth on the basis of its autonomy” (Thompson and Stapleton, 2009, 25; see also Thompson, 2007, Chapter 6; De Jaegher and Di Paolo, 2007). Sense-making, then, is a way of relating to the world and responding to environmental stimuli for the sake of enabling further actions and viability.

The enactive notion of structural coupling captures how organisms relate to their environments. Specifically, coupled systems, such as the honeybee and the flowering plants in its ecological niche, structurally codetermine one another as a result of their reciprocal interactions over time. Varela’s “Bittorio” model was originally conceived to illustrate how such structures co-emerge, though Barandiaran (2017) notes some theoretical difficulties with the model and offers a set of models illustrating the sensorimotor constitution of neurodynamic patterns as a more robust example of how autonomous systems are structurally coupled. On Barandiaran’s account, Bittorio is problematic as an example of structural coupling due to the fact that environmental features are both random and held static. Barandiaran suggests that this is an insufficient characterization of the environment on an enactive framework. An organism’s environmental state-space is not merely a random setting but is constituted by features corresponding to its sensorimotor capacities, and crucially to the enactive approach, organisms are not passive receivers of environmental stimuli but are coupled with the environment in such a way that impacts the structure of the environment. The conceptualization of the environmental state-space in the Bittorio model seems to conflict with one of the key tenets of the enactive approach, namely, that dynamic interactions with the environment shapes which features will affect the system; the environment cannot merely be conceived of as an independent producer of stimuli.

Using Varela’s example of bacteria swimming up a sucrose gradient (Varela, 1991, 1997), Di Paolo (2005) suggests that merely describing the system as autopoietic is not enough to explain the dynamic coupling between the bacteria and the sucrose environment and the interactions between the two systems. More is needed that explains “graded notions such as lacks and breakdowns and articulates in detail how signification is generated” (Di Paolo, 2005, 437). It is not merely the case that the bacteria constitute autopoietic systems, while the sucrose gradient constitutes the environmental state-space. The sucrose has a degree of valence for the bacteria, as suggested by the concept of sense-making. It invites further activity as specifically an action that is dependent upon on the internal state of the bacteria at that particular time. Therefore, there is some further aspect to the coupled system that generates a particular action on behalf of the bacteria:

As defined, structural coupling is a conservative, not an improving process; it admits no possible gradation. If the concentration is enough to keep bacteria viable the latter should be equally – not more – viable in a range of higher concentrations. Even if the current rate of nutrient intake is lower than the rate of consumption (leading to certain loss of autopoiesis in the near future), bacteria will not seek higher concentrations just because they are autopoietic since improving the conditions of self-production is not part of the definition of autopoiesis. Only if they are able to monitor and regulate their internal processes so that they can generate the necessary responses anticipating internal tendencies will they also be able to appreciate graded differences between otherwise equally viable states (Di Paolo, 2005, 437).

Di Paolo introduces the concept of *adaptivity* in order to specify how autopoietic systems maintain homeostasis in the face of environmental perturbations and despite existing far from thermodynamic equilibrium. This aspect of autopoietic systems necessitates that they act in accordance with graded norms of vitality and viability—bacteria generate appropriate responses to the presence of a sugar gradient depending upon the state of their internal processes. This necessitates that environmental features have a particular valence depending upon the internal state of the organism and assuming that the organism has some capacity to engage with the world in such a way that deals with negatively valenced conditions such as lacks and breakdowns (see also Weber and Varela, 2002).

Thus, the environment is specified as a source of both perturbations and assistances according to graded norms, to which the organism can respond provided it both has the sensorimotor capacities to do so and those features have a particular valence that corresponds to an organism’s processes of internal regulation. The enactive concepts of sense-making and adaptivity help to flesh out how organisms, as autopoietic systems, respond to particular features of the environment in the ways that they do.

While this approach helps to specify the environment as a state-space populated by elements that correspond to graded norms relative to particular organisms, there remains the question of what processes are responsible for the coupling of these coupled systems. It is clear that the environmental “information” indicating pollen is in some way coupled with the honeybee’s capacity for sensing that information. However, this suggests something of a synchronic view of dynamically coupled systems—it tells us why an organism may be acting in a certain manner at a certain time. The environment is here specified as an organism’s cognitive domain, but the structure of the environment, itself, is unspecified beyond that of contingent relations with the sensorimotor capacities of individual organisms. Cognition is undoubtedly more complex of a phenomenon than individual instances of perception and action, and so in order to serve as a rich theory of cognition, enactivism, I want to suggest, requires a further fleshing out of the processes relevant to the generation of coupling between systems. In other words, I hold that it is worth investigating the features of the structure of organisms as cognizing systems and the system that makes up their environmental state-space. This task requires looking at the diachronic relations between organisms and their dynamic niches, which will be the focus of the Specifying the Cognitive Domain section.

It is worth noting that early works in the enactive approach, namely, *The Tree of Knowledge* (Maturana and Varela, 1987), did, indeed, give treatment to these biological questions, suggesting that a history of interactions between systems can result in structural selection acting upon those systems, which, in turn, gives way to a particular determination of structure for each system. This evolutionary-scale claim appears in *The Embodied Mind* in the form of “evolutionary path-making,” and is again addressed in *Mind in Life*, under the concept of “enactive evolution.” These arguments are undoubtedly valuable in that they weave additional biological considerations into an enactive account of cognition, thus resulting in a naturalized approach to cognition. However, more recent work in enactivist thinking has, for the most part, put aside these biological considerations, despite the fact that recent developments in evolutionary and developmental biology (Griffiths and Gray, 2001; Stotz, 2014) have potentially useful resources to contribute to the discussion. Therefore, drawing attention to this dimension of enactivist thinking and expanding upon that work can be a fruitful task to take on.

An enactive view of cognition treats it as a phenomenon spanning a range of timescales, from those as short as only a few milliseconds (the domain of neurophysiology) all the way up to an evolutionary timescale (Varela, 1999; Gallagher, 2017). At each scale, the emphasis on dynamic coupling between processes within the larger organism–environment system remains crucial; the enactive treatment of cells, nervous systems, and organisms as each constituting an autonomous system both situated in, and specified by, a particular environmental context illustrates how the approach is applied at various spaciotemporal scales. What remains constant is the dual-system organization; the autonomous system is always specified in the context of an environmental setting. The environment, therefore, can refer to any state-space in which an autonomous system persists. A parasite’s environmental setting is its host; a cell’s environmental setting is a molecular background. At the scale of the individual organism, namely, for medium-sized animals typically under investigation in the study of perception and cognition, the environmental setting is specified as its ecological niche. The environment is the appropriate cognitive domain for an organism, a feature of the enactive approach that ecological psychology shares.

## Perceiving Environmental Information

In contrast to commonplace views of perception that describe it as a process of inferring information from environmental stimuli, ecological views of perception treat it as a means of directly picking up information from the environment. James Gibson’s work, which serves as a canonical approach to ecological psychology, emphasized the direct perception of environmental information. On Gibson’s view, there is no intermediary task for the brain to accomplish in perceiving environmental information, and so there is no need to cognitively represent that information in order to make sense of it. Gibsonian ecological psychology is thus a non-representational account of perception, as is the case with the enactive approach.

### Affordances as Revealed Information in the Environment

On Gibson’s approach, percepts are not representations of objects in the world, but instead are features of the environment itself. These environment percepts are directly sensed by organisms, depending upon their sensorimotor capacities. They inform organisms as to what actions are possible—in other words, what actions are afforded to the organism. Thus, Gibson termed these environmental percepts as *affordances*. Affordances make direct reference to what is physiologically possible for an organism. For the honeybee, the pollen-rich flower affords landing on, the pollen affords collecting, and so on—whatever is afforded to an organism is something that it perceives and can act upon accordingly. Affordances can therefore be thought of as action-guiding cues from the environment (Stoffregen, 2003).

This conceptualization of the environment should sound relatively similar to that put forth by the enactive approach. There is a key distinction to be made. However, Gibsonian ecological psychology suggests that organisms directly perceive information from the environment, making it the case that such information is built into the structure of the environment itself. In *The Embodied Mind*, Varela et al. (1991) assert that the enactive view does not share this conceptualization of the environment, in that they do not hold that perceptual information is “out there” as a static feature of the environment. Rather, on the enactive view, perceptual information is constructed via the structural coupling between organisms and their environments. Thus, there is an important ontological distinction between the two views. For Gibson, affordances exist independently from perceivers who may (or may not) act on them, whereas for enactivists, perceptual information in the environment is effectively “enacted” via sensorimotor engagement with the world. Varela et al. (1991) clarify that “[w]hereas Gibson claims that perception is direct detection, we claim that it is sensorimotor enactment” (Varela et al., 1991, 204). While Gibson did state that affordances are neither objective properties of the environment nor subjective properties of the perceiver but rather somewhere in between (Gibson, 1979), his is not a constructivist view, as affordances are not in essence created in the interaction between perceiver and environmental stimuli but rather are specified as features acted upon in a relevant manner.

According to Gibson, it is possible for perceiving organisms to “pick up” affordances as visual information due to the way in which the pattern of light reaches a perceiver’s eyes. A setting is visually accessible when ambient light creates a particular structure depending on the position of the perceiver. For example, if you are sitting on a garden bench surrounded by trees and a garden table, the angles at which the light from the sun hits these objects will illuminate the setting allowing you to visually perceive your surroundings. Gibson describes this particular kind of visual arrangement as the *ambient optic array*. The geometric structure of this setting is dependent upon the position of the perceiver—the angles at which the light hits the perceiver’s retina will change as the perceiver moves around in the environmental setting. Through movement, an important feature of the optic array is revealed—some features change, such as the particular angles relative to the light source and the perceiver’s location, but some features are invariant. Thus, invariant structure is revealed through movement, in addition to variant structure:

In the optic array, presumably, there is an underlying invariant structure to specify the edges and corners of the layout and the colors of the surfaces, and at the same time there is a changing structure to specify the temporary direction of the prevailing illumination. Some components of the array never exchange places – that is, they are never permuted – whereas other components of the array do. The former specify a solid surface; the latter specify insubstantial shadows only (Gibson, 1979, 89).

The invariant structure of the garden table, for example, is revealed through movement relative to the ambient optic array, and it is this information that can then be acted upon by the perceiver. The ambient array provides structure to the environmental setting in such a way that, in turn, provides visual access to features of that environment.

### Naturalizing Perception and the Problem of Specifying Variables

Gibson’s account of perception appears, at least at first glance, firmly naturalistic, relying upon optics as the means by which we establish perceptual contact with the world, rather than an inferential process dependent upon the construction of a conceptually imprecise notion of representation. Indeed, as Withagen and Chemero (2009) note, Gibson’s approach was an important contribution to the naturalization of perception. Conceiving of perception as a biological function invited discussion of how organisms endowed with a perceptual apparatus made use of the information available to them via that apparatus as well as how they came to be endowed with such—in other words, how and why they evolved the capacity for visual perception. Yet while Gibson’s ecological approach provided a way of talking about perception in a naturalized manner, Withagen and Chemero (2009) suggest that further developments by neo-Gibsonians introduced new problems.

Neo-Gibsonians (Shaw and Kinsella-Shaw, 1988; Turvey, 1990) elaborated on Gibson’s claim that perceptual information in an environment is specified by the structure of that environment. Their work details a specificity relation between the perceptual information and the environmental feature, and a further specificity relation between the organism’s perceptual experience and the perceptual information, making perception “specific to information that is specific to a particular environmental property” (Withagen and Chemero, 2009, 368). On the neo-Gibsonian view, then, there is a one-to-one-to-one mapping between the environment, the perceptual information in the environment, and the perceptual activity. The environment provides the structure for perceptual information to be accessible, and the perceiver is then able to pick up this information through their locomotive behavior in the environmental setting.

While Withagen and Chemero (2009) note that the lawlike generality described by this mapping relation is appealing, especially for a naturalistic framework, empirical concerns arise. They stress that “the one-to-one-to-one theory assumes an absence of variation in what information is exploited both between animals and within animals over time … In other words, all members of a species use the same information in their perception of a particular environmental property” (369). They question whether such a theory is plausible on a naturalistic approach to perception—that is, on one that treats perception as a biological phenomenon that is subject to evolutionary pressures over time. Given evolutionary considerations, they hold that the one-to-one-to-one theory is implausible: the two specificity relations fail to hold empirically under biological scrutiny.

The specificity relation between perception and environmental information suggests that members of the same species, perceptually endowed in the same manner, make use of the same environmental information in their perceptual activity. On this perspective, all honeybees treat UV light as an action-guiding visual cue, while all humans do not. However, Withagen and Chemero (2009) note that this claim is inconsistent with the biological concept of variation. Variation is necessary for evolution to occur, so in any species subject to evolutionary change, variation must be present in order for it to then be acted upon by selection. Suggesting that perceptual information is specified in an exact manner relative to a perceiving population leaves no room for variation, thus making the theory biologically untenable. Individual differences in various traits, including perceptual abilities, must be possible, making it the case that a specificity relation between a specific variable as environmental information and a perceiver is too strict.

Indeed, differences in perceptual abilities only mark one way in which individual variation may influence relations between environmental information and perception of that information. Other psychological and physiological qualities can have a relevant impact on perception–action dynamics as well. Salient examples can be found in Dennis Proffitt’s work on embodied perception: Proffitt (2006) found that distances to targets appeared greater to participants when they were tasked with carrying a heavy backpack. Thus, as Proffitt explains, physiological potential can have a profound impact on perception. This quality not only varies between individuals but there can also be significant within-individual variation.

Differences that arise as a result of perceptual learning help to illustrate the tension in the neo-Gibsonian view. Withagen and Chemero (2009) cite numerous studies that show how perceivers can learn to exploit new perceptual information, and additional research found significant between-subject variation in perceptual learning ability (Withagen and van Wermeskerken, 2009). They assert that this work shows how human perceivers.

Vary in how well and quickly they can learn a perceptual task, implying variation in what information is exploited at any moment in time. Hence, the ubiquitous variation among the members of a species that proponents of Darwin’s population thinking emphasize … is also present in the perceptual realm. This means that population thinking needs to be taken seriously in the study of perception. In other words, the suggested specificity relation between information and perception and the allied search for *the* information that members of a species exploit in a particular perceptual task are biologically unsound (Withagen and Chemero, 2009, 374).

A one-to-one mapping between environmental information as a specific variable and the perception of that information is therefore problematic on a biological basis. Individuals learn how to differentiate between variables in the environment, making it the case that through learning they can act on information that they previously did not make perceptual contact with. In addition, individuals vary in their perceptual abilities (color vision deficiencies are a simple and common example), so mapping species-typical perceptual abilities onto specific environmental variables may result in biological inconsistencies. For a naturalized theory of perception, biological inconsistencies are severely problematic.

With regard to non-human perceivers, similar results have been found. The perceptual learning capacities of insects are commonly studied, namely, in terms of color vision. In many of these studies, insects are found to possess the ability to make visual discriminations after undergoing learning tasks, illustrating the effect of individual experience and learning on visually guided action. Honeybees, for example, are trichromatic, with the capacity for visual discrimination in color space. Avarguès-Weber et al. (2010) found that free-flying honeybees were able to make more fine-grained color distinctions after aversion training, showing how individual differences and learning experiences can change what perceptual information they interact with. The one-to-one mapping between specified values in a color space and perceptual activity suggested by the neo-Gibsonian approach fails to hold in these cases, as perceptual learning opens up the possibility for interacting with new perceptual information in a non-species-typical manner. These individual differences are important on an evolutionary account in terms of looking at possible mechanisms for, in this instance, the evolution of color vision as movement through color space via novel perceptual abilities.

While Gibsonian ecological psychology provides a naturalized approach to perception that is suitable for understanding perception as an evolved capacity, I am in agreement with Withagen and Chemero (2009) that “a naturalistic theory of perception must explain the individual differences in what information is exploited in terms of the interplay of multiple organismal and environmental factors” (379). For this reason, a neo-Gibsonian reading may prove inadequate, and there is a task for researchers working in the Gibsonian tradition to address these biological concerns if the theory is to prevail as a naturalistic approach. Describing environmental, perceptual information as specified variables fails to account for individual differences in perceptual ability, especially due to learning and experience.

Looking beyond the scale of species-typical perceptual ability and instead considering the individual perceiver’s relation to its environmental setting requires, in turn, looking at individual-specific environmental settings. Often an individual organism is represented as an idealized member of its species, and given research constraints, necessarily so. However, such limitations should not stop researchers from looking more closely, and more carefully, at what constitutes an individual’s environmental milieu for the sake of understanding how an idealized individual might relate to that setting. Indeed, as stressed in this section, looking at potential ways in which individual differences may bring about evolutionary change is necessary for understanding biological diversity, and in thinking about perception as a biological phenomenon.

In the next section, I suggest a way forward for how to specify an individual’s cognitive domain for the sake of understanding how environmental information co-varies with perceptual abilities. This approach respects the goal of naturalization of perception as found in Gibsonian ecological psychology, while at the same clarifying the notion of an “enacted” world central to the enactive approach to perception and cognition (see also McGann, 2014 for a complementary approach). In particular, I suggest that it is helpful to think of an individual organism’s cognitive domain as mapping onto its developmental niche, which is the environment in which it undergoes its life cycle. This specification, I argue, provides a way of thinking about the environment in organism–environment systems that is complementary to both enactivism and ecological psychology, while at the same time addressing some of the ambiguities that arise in both fields’ use of the environmental setting as a key part of understanding cognition, action, and perception.

## Specifying the Cognitive Domain

I am not using the term “cognitive domain” in any specific technical sense; it is simply meant to refer to an organism-specific environmental state-space in which cognitive activity takes place. We can think of a bee’s cognitive domain, for example, as the environmental state-space containing whatever is potentially perceivable and actionable by the bee. Whatever those elements are will depend upon the bee’s sensorimotor capacities. On an enactive reading, the bee’s enacted world is its cognitive domain, in virtue of its autopoietic, adaptive configuration. According to ecological psychology, the bee’s cognitive domain is populated by affordances, with certain affordances acted upon according to the bee’s perceptual activity^1^.

Determining what elements populate an organism’s cognitive domain requires careful investigation of its sensorimotor capacities and its coupled history with its environment (the details of which matter will be addressed in the Developmental Niche section). For example, eyes are ubiquitous throughout the natural world. Yet even in locations where vision is seemingly no longer worth investing resources in, such as in subterranean habitats, eye structures, though reduced, persist (Nevo, 1979; Nikitina et al., 2004). Normal development for the naked mole rat (*Heterocephalus glaber*), for example, results in a reduced eye structure, but an ocular phenotype nevertheless. Researchers have asked why this trait still develops despite at least 25 million years of subterranean evolutionary pressures (Bennett and Faulkes, 2000; Nikitina et al., 2004). Just like any organ, eyes have metabolic costs, and so individuals who direct those resources elsewhere may be better off. Nikitina et al. (2004) suggest, however, that a closer inspection of the environmental stimuli and the mole rats’ regular activities can provide clues as to why the phenotype has not been completely selected against:

… Retaining the capacity for light–dark discrimination is important for the survival of these animals. The soil-removal activity of the naked mole rats results in their direct exposure to sunlight, as the animals kick soil out of an open mound. The open mound poses a further threat of exposure to aboveground predators (Sherman et al., 1991). An ability to detect light and dark and sudden transitions associated with the arrival of a predator at a well-lit burrow entrance may confer a survival advantage and hence be maintained by natural selection (Nikitina et al., 2004, 331).

While the species-typical habitat of the naked mole rat is categorized as being distinctly subterranean, brief instances of direct exposure to sunlight is enough to serve as an environmental pressure necessitating the retaining of an eye structure. The payoff of these reduced eye structures is a discounted metabolic cost along with a sufficient capacity for light–dark discrimination. These findings support an account of perception that stresses how perception is actually used—while there are no scientific findings that suggest that the mole rats use visual information in the way animals with fully developed eyes typically do, their eyes still pick up environmental information—specifically, transitions in brightness. They may not be able to “establish perceptual contact” with objects in the world (including conspecifics, which they identify through olfactory and tactile cues) (Nikitina et al., 2004, 331), but in a sense, light still affords them seeing, albeit an unconventional mode of seeing.

This example is meant to show how careful investigation of the specifics of the relationship between perceiving organisms and their environments matters to how we think about perception and action. The mole rats’ habitat is not merely a subterranean one, and the particular way they interact with that environment, even if in brief moments, can end up impacting their evolutionary trajectory. As we saw, occasional surfacing in the activity of burrow building has generated enough selective pressure to retain minimal eye structures. It may not be a conventional way of using eyes, but it works for the mole rats.

Persisting both at the developmental and at the evolutionary scale is often a matter of getting by on what works rather than maximizing potential. Indeed, Varela et al. (1991) note this biological fact in their reference to evolution as natural drift, stating a call for recasting selective pressures as “broad constraints to be satisfied” (Varela et al., 1991, 198). They refer to this satisficing principle in describing the enactive notion of mutual specification, or the Lewontin-inspired notion of codetermination (Lewontin, 1983):

The key point, then, is that the species brings forth and specifies its own domain of problems to be solved by satisficing; this domain does not exist “out there” in an environment that acts as a landing pad for organisms that somehow drop or parachute into the world. Instead, living beings and their environments stand in relation to each other through *mutual specification* or *codetermination*. Thus what we describe as environmental regularities are not external features that have been internalized, as representationism and adaptationism both assume. Environmental regularities are the result of a conjoint history, a congruence that unfolds from a long history of codetermination. In Lewontin’s words, the organism is both the subject and the object of evolution (198–199, original italics).

This coupled history matters, particularly in instances where interacting features are both organisms. Pollinators and angiosperms are typically thought to have a mutualistic relationship, with one organism relying upon the other for its survival and reproductive needs. However, an established coupled history between these organisms matters for their viability. Aizen et al. (2014) note that seemingly mutualistic relationships between a native organism and an invasive organism may result in detrimental effects to the native organism. There is a lack of a coupled, shared history between the two species, resulting in an imbalance in costs and benefits to each. An established relationship matters for how environmental features modify organisms over developmental, behavioral, and evolutionary timescales—features may be exploited (or not) with a variety of effects over these timescales. In addition, importantly, organisms, in turn, modify these features, which results in the generation of new developmental and evolutionary effects, as discussed in the literature on niche construction (e.g., Odling-Smee et al., 2003). So the details of interaction matter, especially for a naturalized account of perception. If we want to understand perception as a biological phenomenon, we need to look at how it is actually used, down to the individual differences and peculiarities such as those seen in the naked mole rat. One approach to investigating the details of interaction is to consider the environment at the scale of the individual; this requires specifying the environment in a more fine-grained manner.

### Multiple Senses of Environment

Brandon and Antonovics (1996) suggest that, in the field of population biology, there are three ways to distinguish between conceptions of the environment for the sake of understanding organism–environment coevolutionary dynamics. These conceptions differ based on what sets of environmental factors are taken to be relevant in the generation of selective pressures. The first sense of environment is purely external—the environment is constituted by a set of factors independent of an organism of interest and are measured independently from an organism of interest. Brandon and Antonovics (1996) suggest that a fundamental problem with conceptualizing the environment in this manner, however, is that because these factors are measured entirely independently from the organism, they may turn out to be irrelevant to an organism’s fitness, and thus do no work toward an understanding of how and why populations evolve. So a conception of the environment that merely identifies the set of physical factors external to organisms is insufficient.

The second conception of the environment is the ecological environment, which utilizes organisms as “measuring instruments” (Brandon and Antonovics, 1996, 164) to determine the external factors as they affect population growth. This conception effectively picks out features of the environment that are relevant to a particular lineage such that identifying them sheds light on that lineage’s evolutionary trajectory (Griffiths and Gray, 2001). Brandon and Antonovics (1996) note that a further step is needed in order to compare fitnesses of different genotypes. A third conception of the environment as selective allows for comparison between genotypes in relation to pressures from the environment. As the goal with this approach is to be able to measure organism–environment coevolution by way of assessing organism-relative factors, specifying an environment as a narrower set of features can aid in understanding why some genotypes fare better than others. The selective environment, then, is the appropriate sense of the environment to consider when comparing individual genotypes, and thus individual differences that may over time be selected either for or against.

One way to further parse out these differing senses of environment is in a developmental context, albeit one with relevant evolutionary implications. Each sense of environment can be said to be constituted by a set of resources organisms can make use of, and some of which are necessary for the transgenerational stability of form (Griffiths and Gray, 1994). Identifying the structure of resources available to organisms is a central goal of developmental systems theory (Oyama, 1985). By specifying individual domains with unique sets of resources, it is possible to identify developmental resources, which may arise during the interaction of organisms as developmental processes and the environments in which they are situated. For example, persistent resources may be those specified by the notion of an external environment, such as temperature, gravity, and light. These resources play a role in the developmental process and may potentially be relevant to organismal fitness, but they are not identified with reference to a particular organism and exist independently and regardless of any organismal interaction. The sets of resources specified in more fine-grained scales are all organism-dependent and are thus factors in the ecological sense of environment. Resources specified at an even finer-grained scale can arguably fit into the sense of a selective environment. The availability of these resources highlights interactions that take into consideration individual differences in behavior.

Organisms, as developmental processes themselves, make use of resources across each domain depending on a shared history of interaction and with regard to individual needs. What is relevant for the sake of providing an evolutionary explanation is identifying recurrent interactions between resources and organisms with the capacity to utilize those resources. Variation arises when resources are utilized in a new manner, when new resources are introduced, or existent resources are removed, the relationship with resources is altered, and so on. In this way, the developmental system is comprised of not solely an individual organism interacting with an environment over the course of its life cycle, but rather it extends over both the organism as a developmental process and the developmental resources with which it is coupled such that interactions with those resources constitute a species-typical life cycle. This picture places greater emphasis on the environment, itself, in understanding the life activity of the organism than traditional accounts of ontogeny do, as developmental resources (potential or actual) are integral to the specification of the system as a developmental system. Without reference to these features, the resources available for explaining the transgenerational stability of organismal form are impoverished. Specifying the environment and building that specification into an understanding of organisms as developmental processes embedded within a larger developmental system results in a richer account of ontogeny, with greater explanatory power across a developmental timescale, but also a behavioral and, on a population scale, an evolutionary one.

In ecological psychology, similar attempts have been made to parse out different senses of the environment. Baggs and Chemero (2019) distinguish between the physical world, a species habitat, and an individual organism’s *umwelt*. Here, I think it is helpful to map this distinction onto the three-way distinction between senses of the environment described by Brandon and Antonovics (1996). The physical world approximately corresponds to the notion of an external environment—it is not specified in relation to any particular organism. The sense of the environment as a habitat is species specific and contains affordances as resources typical for that species. The third sense of environment, the umwelt, references Jakob von Uexküll’s concept of a particular organism’s lived environment (von Uexküll, 2010); it is a behavior setting that is “shaped by the places where that individual dwells, and by the history of interactions that the individual participates in” (Baggs and Chemero, 2019, 16). An individual organism’s umwelt, then, references its unique abilities and experiences to determine which features of the world are especially salient to it given these properties.

The goal in introducing this three-way distinction between the physical world, a habitat, and an umwelt is to resolve tensions in Gibson’s original distinction between a perceiver-independent physical world and an affordance-containing yet ambiguous surrounding environment, which roughly corresponds to the notion of a habitat on Baggs and Chemero’s three-way distinction. What the sense of an umwelt is meant to do, in this context, is specify exactly how individual differences result in different affordance spaces. A species-specific habitat contains environment features that are utilized in a species-typical fashion, thus referencing an idealized member of that species. The bee orchid (*Ophrys apifera*) that successful tricks male bees into thinking they are encountering female bees presumably tricks all male bees, but the one male bee that does not fall for this trick does not act on the affordance in a species-typical manner. The world, to this clever bee, appears differently—there is no female bee to encounter, only an equally clever orchid plant. Thus, an umwelt, as an individual-specific, third sense of environment, allows for individual variation, which as Withagen and Chemero (2009) note, is essential for an understanding of evolution on a naturalized account of perception.

### The Developmental Niche

The sense of the environment as an individual-specific environment, I want to suggest, can be built upon by further specifying how structural features of the individual-specific environmental milieu both shape and are shaped by coupled features of the individual organism. This task requires looking at the environment, understood as an organism’s cognitive domain, as an ontogenetic or developmental niche^2^. Individual variation in perceptual activity can be investigated in relation to the developmental niche in which individual organisms live. I want to suggest that specifying the cognitive domain in which organisms are situated as their own developmental niches provides a framework for understanding the environment in such a way that builds on both enactive and ecological cognitive science.

West and King (1987) [see also Stotz (2014)] suggest that the concept of an ontogenetic niche can aid in identifying the set of developmental resources that an individual inherits in addition to genes. The social, cultural, and ecological circumstances that an organism is born into play a prominent role in its developmental trajectory. For example, West and King (1988) [see also Smith et al. (2000)] found that the presence and response of female cowbirds had a significant effect on male song development. Identifying this social influence as a parameter in the male cowbird’s ontogenetic niche guides the understanding of what factors are relevant in the species-typical development of singing behavior. This influence on song learning and development can have transgenerational effects, making it the case that the multimodal (both visual and auditory) sensory feedback from social interactions can serve as an inherited resource.

Griffiths and Stotz (2018) describe a developmental niche as the “set of parameters that must be within certain bounds for an evolved life to occur (or, in more traditional terms, for the organism to develop normally” (Griffiths and Stotz, 2018, 237). Importantly, they distinguish between a developmental niche and the selective niche described by niche construction theory, which they define as “the set of parameters that determine the relative fitness of competing types in the population” (ibid.; see also Stotz, 2017). While the selective niche picks out elements that generate selective pressure on an organism, the developmental niche picks out elements that are relevant for the species-typical development of an organism.

The developmental niche is part of the larger developmental system; it identifies the environmental setting or context in which a developmental system constructs a life cycle. It is the set of parameters that “play a role in the modification and reproduction of the life cycle” (Stotz, 2017, 2). The relevant parameters may be not just physical resources but also “social, ecological and epistemic” (ibid.) resources that aid in the reliable reconstruction of a life cycle (in other words, an individual organism^3^). These resources are inherited in the reconstructing of a life cycle. The claim that extragenetic resources are inherited within the context of a developmental system is a key aspect to DST and differentiates it from traditional accounts of ontogeny.

One example in this regard is Gottlieb’s (1985, 2002) experimental work on duckling vocalization behavior (Gottlieb, 1985, 2002; see also Gottlieb, 2001). While this behavioral trait is typically considered to be instantiated by innate mechanisms, Gottlieb found that particular external factors, such as a duckling’s experience hearing its own vocalizations as well as vocalizations of its siblings while still at the embryotic stage of development, played a significant role in the species-typical development of that behavior (Gottlieb, 2002). This example shows that even something as precise as individual experience, at a very specific stage in development, can affect an organism’s developmental and behavioral (and potentially evolutionary) trajectory:

The intricacy of the developmental causal network revealed in these experiments proved to be striking. Not only must the duckling experience the vocalizations as an embryo (the experience is ineffective after hatching), the embryo must experience *embryonic* vocalizations. That is, the embryonic vocalizations change after hatching and no longer contain the proper ingredients to tune the embryo to the maternal cell (Gottlieb, 2002, 170).

In this sense, an individual organism’s developmental niche is its own unique environmental setting, morphed by its interaction with resources within the niche just as those resources impact it. Whether or not species-typical phenotypes are exhibited is dependent upon specific kinds of interaction between an organism and the resources within its developmental niche. Changes in interaction potentially have a generative effect over time. Shifts in developmental niche are possible through variation in behavior.

In Oyama’s, *The Ontogeny of Information*, organisms are conceived of as integral parts of a larger developmental system, which contains environmental resources that act on and are acted upon by the organism in that system. The developmental system is comprised of a complex web of interactions that impact how the organism develops and changes over its lifetime. In this context, an organism’s developmental niche can be thought of as the specific environmental setting that is comprised of inherited developmental resources part of a larger developmental system. Thinking of the environment as an individual organism’s developmental niche makes it clearer how organisms form certain relationships with certain environmental elements (including conspecifics) and how those relationships can change (and new ones created) over developmental, behavioral, and evolutionary time. This conceptualization leaves room for the creation of new coupling processes via individual innovation, potentially leading to new features of both the organism and its environment.

As Gottlieb stresses, developmental systems are dynamic and in constant flux, with new iterations (i.e., new generations) impacted by prior individual variations acted upon by selection over time. This view of the environment thus avoids potential issues with circularity that may arise if the improper environmental scale is considered. The concern here is that on a generalized account of the environment, which as stressed in the previous section leaves no room for variation, a perceiver–environment system is markedly circular—perceivers pick up relevant environmental stimuli, and environmental stimuli is present as a resource for perceivers. This picture does not tell us why the coupling has arisen or why it persists. Bees perceive UV light, and UV light is perceivable by bees. However, this was not always the case; bees did not pop into existence ready to utilize UV light as an action-guiding visual cue. In a similar vein, one hypothesis for the evolution of trichromatic color vision in some primates was the ability to pick out colored fruit (Allen, 1879). A generalization of this coupling does not tell us how organisms move, evolutionarily, through color space. It properly identifies a coupled system, but provides only a synchronic account of that phenomenon. If we are convinced that a dynamic approach to understanding perceptual activity is a fruitful way forward, we must look at the environmental setting in which perception occurs across multiple timescales—developmentally, behaviorally, and evolutionarily. I have attempted to illustrate that the biological resources for looking at the individual organism across these scales (both spatial and temporal) are plentiful, and thus, a robust naturalized account of perception ought to make good use of them.

In accordance with DST’s concept of a developmental niche and Baggs and Chemero’s sense of the environment as an individual-specific umwelt, I suggest that one fruitful way of specifying the cognitive domain is to characterize it as an individual-specific developmental niche. On this view, conceptual tools from enactivism and ecological psychology can be put to use alongside conceptual tools from DST to result in a cohesive framework for understanding the cognitive domain for perceiving organisms.

Enactivists speak of the “enacted world” of a perceiver as emerging from perceptual interactions contingent upon sensorimotor capacities. The enacted world is populated by environmental features with potential valence to an organism depending on the internal needs of the organism at a specific time (Thompson, 2007; Di Paolo et al., 2017). The enactive approach is thus a naturalistic one, as it draws on the biological factors involved in cognition for explanatory purposes. However, exactly what features populate an organism’s enacted world is dependent upon not only a history of coupled interactions between its species and the ecological environment but also between an individual and its developmental niche. Importantly, these interactions are dynamic, with some couplings strongly conserved over time (such as eye structures and light stress) (Nilsson, 2009; Oakley and Speiser, 2015) and others in flux during an individual’s life cycle (such as differentiation in abilities enabled by learning). Thus, the cognitive domain of a perceiving organism shapes and is shaped by that organism’s influences on various timescales, making the enacted world a dynamic one emerging out of a complex web of interactions as a result of both individual experience and innovation^4^ as well as species-typical behavior.

From ecological psychology, resources across each environmental domain, from persistent resources to self-generated resources (Griffiths and Gray, 1994), can be thought of as affordances in that they invite certain interactions that have the potential to alter both the developmental trajectory of an organism and its species’ evolutionary trajectory. In this way, affordances are conceived of as non-specifying features, in that they vary as a result of cycles of interactions within the developmental system. The problem neo-Gibsonians face due to their commitment to the specification of features, then, is avoided. This leaves room for the evolution of affordances as resources themselves, as well, as repeated interactions with a resource may result in evolutionary change within that resource, as is seen in relationships of coevolution between two species. Variations in interactions, therefore, lends to the possibility of new resources being utilized in the reconstruction of the developmental process, resulting in changes to organismal form over time—in other words, to evolutionary change. Enough repeated iterations of an interaction between a developmental resource and the organism as a developmental process can result in the emergence of new features to make better use of that resource.

Conceiving of the organism-specific environment as a developmental niche can potentially aid in alleviating some ontological tensions between enactivism and ecological psychology. On an enactive reading, the niche is partially constructed by the organism that occupies it and is continuously shaped by the organism’s behavior. According to ecological psychology, the niche is populated by affordances, which exist as physical features of the environment but afford certain actions in relation to the organism’s capacities. Affordance spaces might be constructed by individuals in a literal sense, but they are still features of the environment that can, in turn, have an effect on other systems that occupy that space. For example, a beaver dam is constructed by individual beavers, yet the structure itself can change the flow of the river, can provide a living place for other organisms, and so on. The constructing of an affordance space does not merely change the actions afforded to individual beavers, but has a global ecological effect as well. Therefore, thinking of an affordance space as relational only to the perceivers that are foremost responsible for its construction might result in overlooking some important ecological aspects of that affordance space as an ecological niche.

Importantly, the developmental niche need not be thought of as being populated solely by biological or ecological factors^5^. Social and cultural affordances play a large role in guiding action for humans (Rietveld et al., 2013) and arguably for non-human animals as well (Avital and Jablonka, 2000). The resources an organism inherits in a niche include both physical resources such as food and shelter but also the potential for social interaction with conspecifics and behavioral traditions such as those seen in West and King’s cowbirds. Individuals inherit a species-typical affordance space, but continue to shape it over time via their own behaviors and in regard to their own interests. Although beyond the scope of this paper, the close investigation of the social and cultural affordances in an individual’s developmental niche may reveal valuable insights about individual variation and change.

By specifying the cognitive domain as an individual’s developmental niche comprised of developmental resources, and as an integral part of the larger developmental system, it is possible to gain a better understanding of why perceiving organisms perceive the sort of features that they do, and how they are able to act on perceptual information in the way that they do. Importantly, this account provides us with a way of looking at how novelty, such as the move from dichromacy to trichromacy, may have been generated as a result of complex interactions between organisms and features within their environment. However, it also suggests the need for a complementary psychological view that emphasizes the dynamic relationship between organisms and their environments, across multiple spatial and temporal scales, and it is here that I think enactivism and ecological psychology equally have resources to contribute.

## Concluding Remarks

In their 2019 paper, “Von Uexküll Revisited: Addressing Human Biases in the Study of Animal Perception,” Caves et al., 2019 suggest that human perceptual biases have skewed experimental methodologies in sensory ecology, resulting in inaccurate portrayals of the visual world of differing species. A way forward for sensory ecology, they suggest, is to consider the specific context relevant to the perceptual phenomenon under investigation—that is, to look at which features of the visual environment are salient to an individual of that species, their physiological makeup, their behavioral traits, and so on. An account of perception that looks more carefully at the relationship between organisms and their environments can aid in avoiding such a bias, as it would involve taking seriously how the organism’s body plays a role in its perceptual activity, how certain environmental features are perceptually salient depending on sensorimotor capacities, how organisms directly pick up information in the environment without the need for neurological machinery to translate that information from internal representations, and so on. Such an account would not take for granted how perception is utilized in the natural world. Both enactivism and ecological psychology have the conceptual tools to contribute to this view of perception; both stress the active exploration of the environment as central to understanding perception. However, we must look more carefully at that active exploration, over developmental, behavioral, and evolutionary time, and in turn, we must look at precisely what is being explored in order to understand perception as a biological phenomenon.

In this paper, I have argued that specifying the cognitive domain as an individual-specific developmental niche serves as a way to define the sort of environment that is referred to in the concept of organism–environment systems. This concept of the environment picks out the unique and dynamic relationships between perceiving organisms and their environments that might otherwise go unnoticed on either a physical environment reading or potentially even an ecological environment reading, which focuses on idealized members of a species. Sharpening the explanatory picture in this way allows us to account for individual variation, in line with concerns raised in Withagen and Chemero (2009), and provides a better sense of what generates novel traits by looking at why an individual might either respond differently to existing environmental stimuli or cope with new environmental perturbations by generating novel adaptive responses. As shown in the previous section, these insights can shed light on broader questions about perception, and in such a way that shows the advantages to an account of perception that investigates the organism as a whole in the context of its surroundings. Both enactivism and ecological psychology share that commitment, and thus, the hope is to bolster both theories simultaneously by appealing to such a framework.

## Author Contributions

The author confirms being the sole contributor of this work and has approved it for publication.

## Conflict of Interest

The author declares that the research was conducted in the absence of any commercial or financial relationships that could be construed as a potential conflict of interest.

## Footnotes

1 I am aware that there may be a tension here, as affordances are typically understood as existing independently from a perceiver, and on this account, it seems as though only organism-specific affordances populate its cognitive domain. One way to ease this tension might be to suggest that the specification of a cognitive domain is meant to serve as a heuristic tool. In other words, specifying an organism’s cognitive domain can be helpful in determining what might be cognitively relevant to it, but it does not have to entail that the organism is limited to perceiving only those elements.

2 While West and King use the term “ontogenetic niche,” Griffiths and Stotz note that they use “developmental niche” as a synonym in their work (Griffiths and Stotz, 2018; see also Stotz, 2008, 2010; Griffiths and Stotz, 2013). I will use “developmental niche” here to make it clear that I am drawing mainly from DST.

3 One route to pursue as an application of this framework is the task of identifying what qualifies as an organism in organism–environment systems. I do not have the space to give this task adequate treatment here, but I do think there are resources available for addressing it. For example, we might think of the organism in the organism–environment (or developmental) system as the process of an individual life cycle (see Griffiths and Gray, 1994, 2001; Griffiths and Stotz, 2018). Recent discussions on process ontology in biology (see Nicholson and Dupré, 2018) are also resourceful. A process view of the organism would fit fairly naturally with the enactive approach, I think, though I am less sure of how well it would connect with ecological psychology. I thank an anonymous reviewer for drawing attention to this important question.

4 One example of (often individual) innovation having a downstream effect is the notion of “cumulative culture,” or the “ever-increasing, additive complexity or efficiency of cultural performance over time” (Schofield et al., 2018). The sweet potato washing behavioral repertoire of Japanese macaques began with a single innovator, with the behavior quickly spreading to others in the group. It is noted that the washing has the effect of reducing parasitic infections, thus suggesting an adaptive aspect to the behavior as well.

5 Many thanks to an anonymous reviewer for highlighting the relevance of social affordances in this context.
